# Scutellarin-induced A549 cell apoptosis depends on activation of the transforming growth factor-β1/smad2/ROS/caspase-3 pathway

**DOI:** 10.1515/biol-2021-0085

**Published:** 2021-09-07

**Authors:** Guang-Yan Zhang, Wei-Yong Chen, Xiao-Bo Li, Hua Ke, Xue-Lin Zhou

**Affiliations:** Respiratory Department, The Chengdu Seventh People’s Hospital, Wuhou District, Chengdu, Sichuan 610000, People’s Republic of China

**Keywords:** scutellarin, A549 cells, TGF-β1/smad2, ROS, cleaved caspase-3, apoptosis

## Abstract

Scutellarin plays an anti-tumor role in A549 lung cancer cells, but the underlying mechanism is unclear. In this study, scutellarin was used to treat A549 cells for 12, 24, and 48 h, followed by the addition of Tempo, a selective scavenger of mitochondrial reactive oxygen species (ROS) and SB431542, a transforming growth factor (TGF)-β1 receptor inhibitor. A dihydroethidium fluorescence probe was used to measure the intracellular ROS level, Cell Counting Kit-8 (CCK-8) was used to detect cell viability, and flow cytometry was performed to examine apoptosis. Western blots were used to detect the total protein level of TGF-β1, p-smad2, and cleaved caspase-3 in A549 cells. The results showed that scutellarin significantly inhibited cell viability and increased apoptosis. Scutellarin also promoted intracellular ROS production, TGF-β1/smad2 signaling pathway activation, and cleaved caspase-3 expression, which was partly reversed by Tempo. Moreover, scutellarin-induced intracellular ROS production and cleaved caspase-3 expression were inhibited by blocking the TGF-β1/smad2 pathway with SB431542. In conclusion, scutellarin promoted apoptosis and intracellular ROS accumulation, which could be abrogated by Tempo and SB431542 treatment in A549 cells. Our study indicated that scutellarin induced A549 cell apoptosis via the TGF-β1/smad2/ROS/caspase-3 pathway.

## 1 Introduction

Lung cancer is diagnosed in 1.04 million cases and causes 921,000 deaths every year in China [1]. Non-small cell lung cancer accounts for 85% of all lung cancer cases and is the most common type [2]. At present, although methods such as chemotherapy and radiotherapy have been partially successful, lung cancer has still not been cured.

Scutellarin, extracted from the perennial herb, is a flavonoid with a free hydroxyl in the 7 position [3]. One study reported that in malignant glioma, breast carcinoma, and prostate cancer, scutellarin inhibited cell viability and increased the apoptosis rate [4]. Guo et al. reported that scutellarin generated mitochondrial reactive oxygen species (ROS) which resulted in apoptosis of human colon cancer HCT116 cells [5]. Moreover, an inhibitory effect on cell proliferation was observed following scutellarin treatment of lung cancer cells [6]. However, the underlying mechanism remains unclear.

It was reported that scutellarin promoted the production of ROS which selectively reduced the survival rate of multiple myeloma cells and induced apoptosis without affecting non-malignant cells [7]. Das et al. demonstrated that transforming growth factor (TGF)-β1 induced oxidative stress to generate ROS production through a mitochondrial-dependent pathway and induced cell apoptosis via cleavage of caspase-3 [8]. TGF-β is a secreted growth differentiation factor that binds to TGF-β receptor II and recruits and phosphorylates TGF-β receptor I [9]. Activated TGF-β receptor I phosphorylates two different smad proteins in the case of TGF-β, smad2 and smad3 [10]. These smad proteins then interact with smad4 to form the oligomeric complexes smad2/smad4 [11] and Smad3/Smad4 [12], which translocate to the nucleus, bind DNA, and regulate transcription. Because TGF-β has potent growth inhibitory activity in a variety of cells, it is considered a tumor suppressor [13]. The TGF-β/smad signaling pathway plays important roles in cancer cell differentiation, proliferation, and apoptosis [14]. The activation of the TGF-β/smad signaling pathway mediates apoptosis, contributing to its anti-oncogenic effect in lung cancer, which is supported by studies showing that TGF-β1 plays an important role in mediating apoptosis of small cell lung cancer cells [15] and that the TGF-β1/smad2 signaling pathway is responsible for dexamethasone-induced apoptosis of human lung A549 adenocarcinoma cells [16]. Therefore, we hypothesized that the TGF-β/smad2 signaling pathway may be responsible for A549 cells’ apoptosis, which is also induced by scutellarin.

In our study, Cell Counting Kit-8 (CCK-8) proliferation assays were performed to detect the cell viability of scutellarin-exposed A549 cells. Annexin/propidium iodide (PI) staining was used to detect apoptosis of A549 cells. A dihydroethidium (DHE) probe was added to scutellarin-exposed A549 cells to quantify the intracellular ROS levels. Western blots were then performed to detect the protein levels of cleaved caspase-3, TGF-β1, and phosphorylated (p)-smad2. The results demonstrate that scutellarin induces A549 cell apoptosis through the TGF-β1/smad2/ROS/caspase-3 pathway.

## 2 Materials and methods

### 2.1 Cell culture and reagents

Dulbecco’s modified Eagle’s medium with 10% of fetal bovine serum (Thermo Fisher Scientific, Waltham, MA, USA) was used to culture A549 cells (American Type Culture Collection, Manassas, VA, USA). When the confluency of A549 cells reached 80–90%, the cells were passaged. CCK-8 reagent was purchased from the Tongren Institute of Chemistry (Dojindo, Japan). Primary antibodies against GAPDH, TGF-β1, p-smad2, smad2, and cleaved caspase-3 (all diluted 1:1,000) were purchased from Abcam (Cambridge, MA, USA). Scutellarin, Tempo, and SB431542 were obtained from MedChemExpress (Monmouth Junction, NJ, USA).

### 2.2 CCK-8 proliferation assay

The cell viability of A549 cells was tested in CCK-8 proliferation assays. In short, A549 cells were inoculated in 96-well microplates at a concentration of 1 × 10^4^ cells/well, incubated at 37°C with 5% of humidified CO_2_, then cultured in medium with and without scutellarin for 12, 24, and 48 h, followed by addition of CCK-8 reagent and incubation at 37°C for 1 h. A miniature flat panel reader (BioTEK, Winooski, VT, USA) was used to detect absorbance at 490 nm.

### 2.3 Annexin/PI staining

Flow cytometry (Annexin/PI staining) was used to detect the A549 apoptosis rate. In short, about 1 × 10^5^ cells were harvested and washed twice with PBS. Binding buffer (100 μL) containing Annexin V-FITC and PI (Beyotime, Shanghai, China) was added to stain the cells. A FACScan flow cytometer (BD Biosciences, Franklin Lakes, NJ, USA) was used to quantify Annexin V-FITC and PI staining on channels FL-1 and FL-3, respectively, and CellQuest Pro software (BD Biosciences) was used for analysis.

### 2.4 Detection of ROS levels by fluorescence microscopy

A DHE fluorescence probe (DCFH-DA; Beyotime, Shanghai, China) was used to determine the level of intracellular ROS. In short, the cells were treated with DCFH-DA (10^−5^ M final concentration) and incubated in a light-protected humidification chamber at 37°C for 30 min and then washed with PBS. In this experiment, the excitation wavelength (488 nm) and emission wavelength (525 nm) were set by the fluorescence microscope (Axio Observer Z1Magol, Carl Zeiss, Oberkochen, Germany). The fluorescence intensity was quantified by Image-Pro Plus version 6.0 software (Media Cybernetics, Rockville, MD, USA) to compare the results between groups.

### 2.5 Western blotting analysis

The cells were treated with cleavage buffer containing protease and phosphate inhibitors (Beyotime) for 30 min. The lysate was collected and centrifuged at a speed of 12,000 rpm at 4°C for 15 min. The supernatant was collected and boiled with loading buffer (Beyotime). Cell proteins (30 μg) were subjected to SDS-PAGE in 10% of gels and transferred to polyvinylidene fluoride membrane. The blots were blocked in 5% of skimmed milk, incubated with the primary antibody overnight at 4°C and then incubated with the secondary antibody for 2 h at room temperature. The immunoreactive proteins were visualized by chemiluminescence.

### 2.6 Statistical analysis

Data were expressed as mean values ± SD from three independent experiments. Statistical significance between groups was assessed by one-way analysis of variance followed by Tukey’s test and Student’s *t*-test using Prism 5.01 (GraphPad Software, San Diego, CA, USA). A *P*-value <0.05 was considered statistically significant.

## 3 Results

### 3.1 Proliferation decreased and apoptosis increased in scutellarin-exposed A549 cells

**Figure 1:**
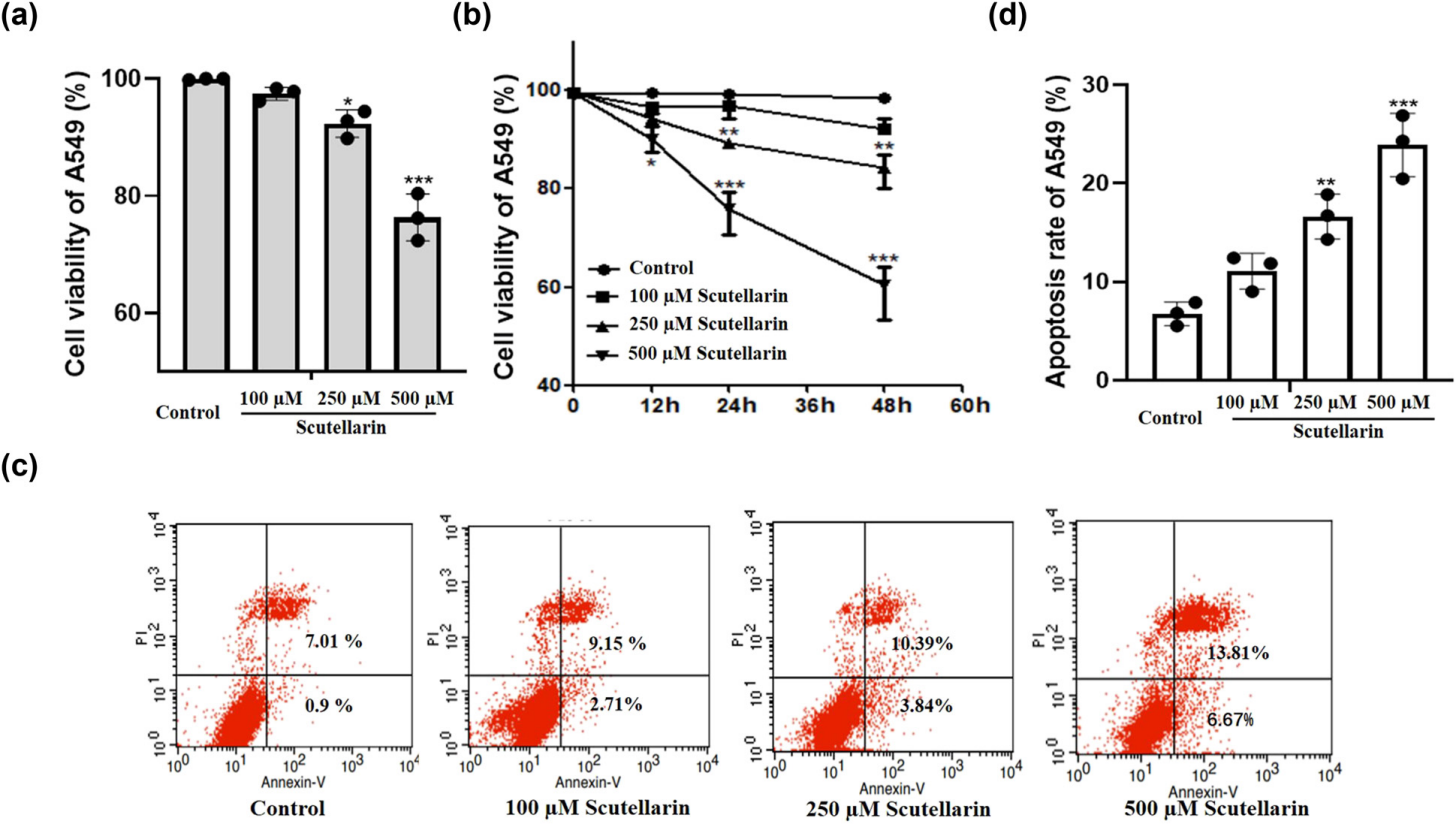
Proliferation was decreased and apoptosis was increased in A549 cells by scutellarin treatment. (a) A549 cells were exposed to scutellarin at a concentration of 0 (control), 100, 250 and 500 µM for 48 h and then CCK-8 assays were performed to assess cell viability. (b) A549 cells were exposed to scutellarin at a concentration of 0 (control), 100, 250 and 500 µM for 12, 24, and 48 h and then CCK-8 assays were performed to assess cell viability. (c and d) A549 cells were exposed to scutellarin (500 µM) for 24 h and then the apoptosis rate was tested by flow cytometry. *P < 0.05, **P < 0.01, and ***P < 0.001.

A549 cells were exposed to scutellarin (Control (0), 100, 250 and 500 μM), and cell viability was detected by CCK-8 assays at 48 h. With the increase in scutellarin concentration, the proliferative activity of A549 cells gradually decreased, which indicated that scutellarin decreased A549 cell proliferation in a concentration-dependent manner (Figure 1a). We also observed that scutellarin reduced A549 cell proliferation in a time-dependent manner (Figure 1b). The Annexin/PI staining results showed that the apoptosis rate of the scutellarin-treated group was higher than the untreated group (Figure 1c and d).

### 3.2 Intracellular ROS levels and expression of TGF-β1, smad2, and cleaved caspase-3 increased in response to scutellarin exposure

**Figure 2:**
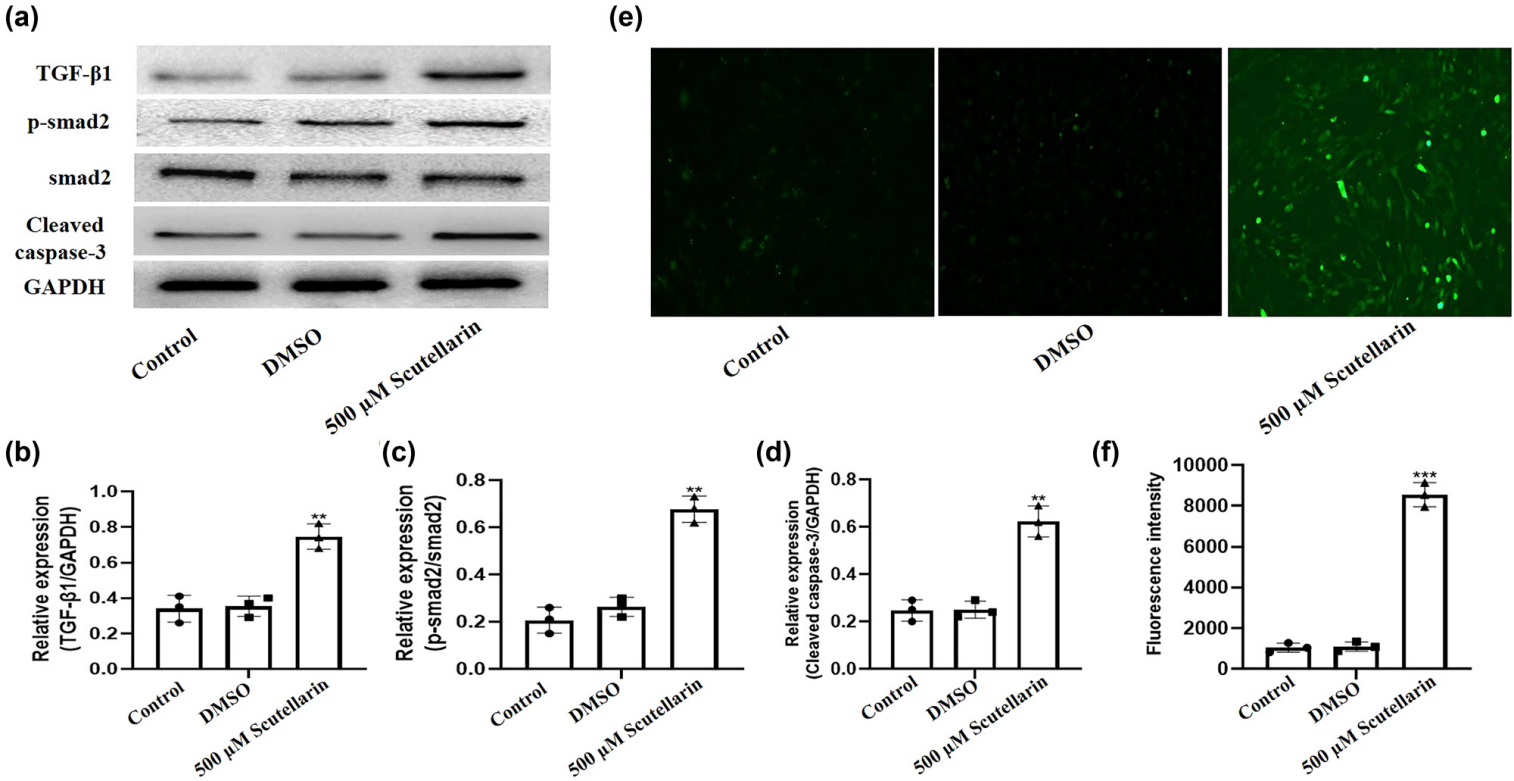
Intracellular ROS level and expression of TGF-β1, smad2, and cleaved caspase 3 in A549 cells were increased after scutellarin exposure. A549 cells were treated with 500 µM scutellarin for 48 h. (a–d) Western blots were prepared to detect the expression of TGF-β1, p-smad2, smad2, and cleaved caspase-3, and the results were analyzed by Prism version 5.01 software. (e and f) The intracellular ROS level was detected using a DHE fluorescence probe and then analyzed. **P < 0.01 and ***P < 0.001  vs control.

To explore the effect of scutellarin on the protein level of TGF-β1, smad2, and cleaved caspase-3 in A549 cells, the cells were treated with 500 µM of scutellarin for 24 h. It was observed that scutellarin promoted the expression of TGF-β1 and p-smad2 (Figure 2a–c). Caspase-3 is a key apoptotic molecule which is activated in the early stage of apoptosis. Activated caspase-3 consists of two large subunits (17 kDa) and two small subunits (12 kDa) which are cleaved by the corresponding cytoplasmic and nuclear substrates and eventually lead to apoptosis, which was measured after scutellarin treatment. Scutellarin significantly increased the expression of cleaved caspase-3 (Figure 2d). The intracellular ROS level was detected by DHE fluorescence probe and was significantly increased by scutellarin (Figure 2e and f).

### 3.3 Scutellarin-induced intracellular ROS level and cleaved caspase-3 expression were inhibited by Tempo

**Figure 3:**
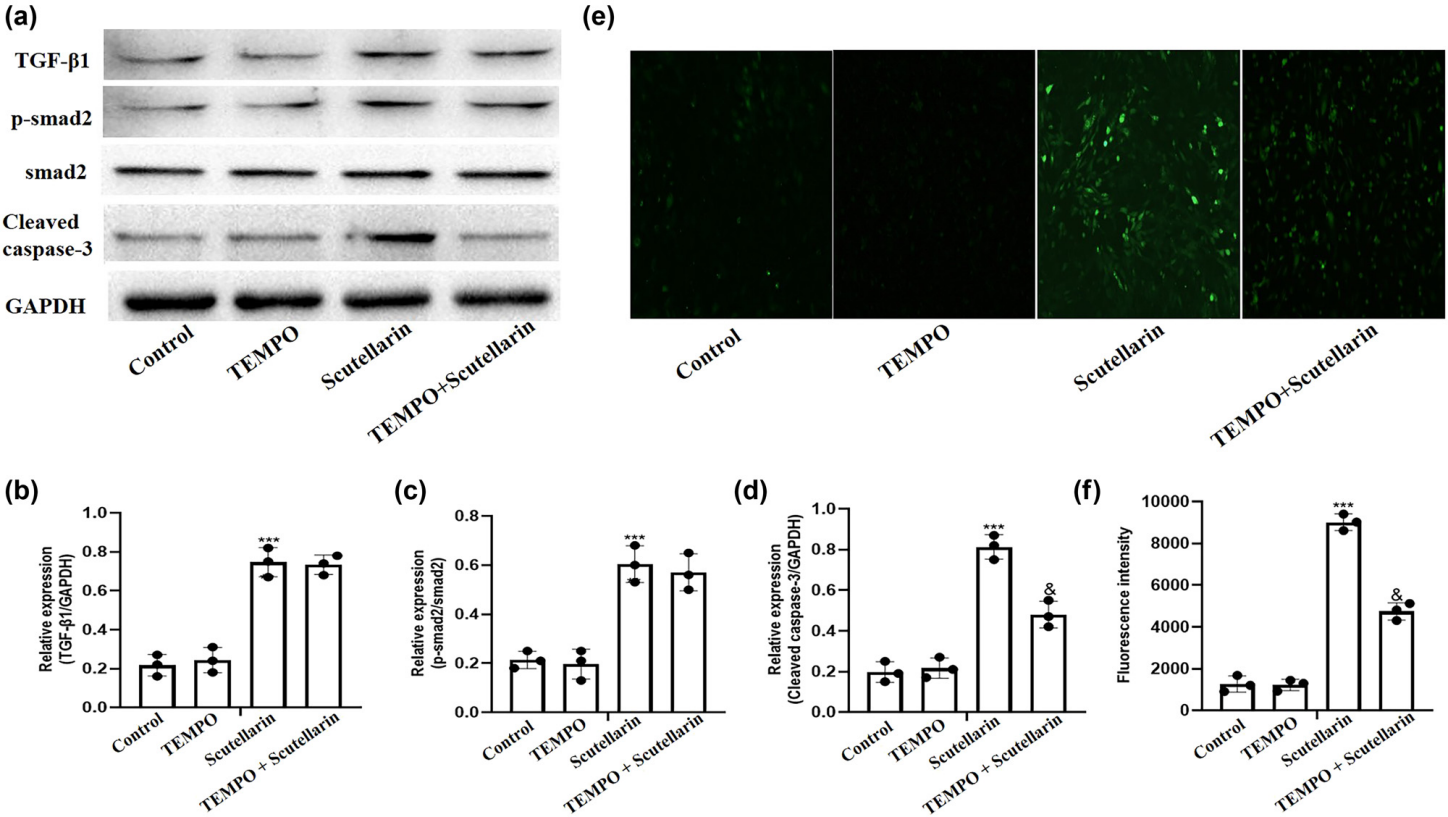
Intracellular ROS level and expression of cleaved caspase-3 induced by scutellarin were reversed by Tempo. (a–d) A549 cells were exposed to 500 µM of scutellarin with and without 100 µM of Tempo for 48 h, and western blots were prepared to detect the protein level of TGF-β1, p-smad2, and cleaved caspase-3. (e and f) The intracellular ROS level was detected using a DHE fluorescence probe and then analyzed. ***P < 0.001 comparing the scutellarin + Tempo group vs the scutellarin group.

In order to explore the mechanism of apoptosis in scutellarin-treated A549 cells, we treated A549 cells with 500 μM of scutellarin and then added 100 μM of TEMPO, a selective scavenger of mitochondrial ROS. Western blots were then performed to detect the expression of TGF-β1, p-smad2, smad2, and cleaved caspase-3. TEMPO treatment significantly inhibited scutellarin-induced caspase-3 cleavage without affecting TGF-β1, p-smad2, or smad2 expression (Figure 3a–d). The intracellular ROS level was then detected using the DHE fluorescence probe and analyzed. ROS production induced by scutellarin exposure was inhibited by Tempo treatment (Figure 3e and f).

### 3.4 Intracellular ROS levels and apoptosis in scutellarin-exposed A549 cells were inhibited by blocking the TGF-β1/smad2 pathway with SB431542

**Figure 4:**
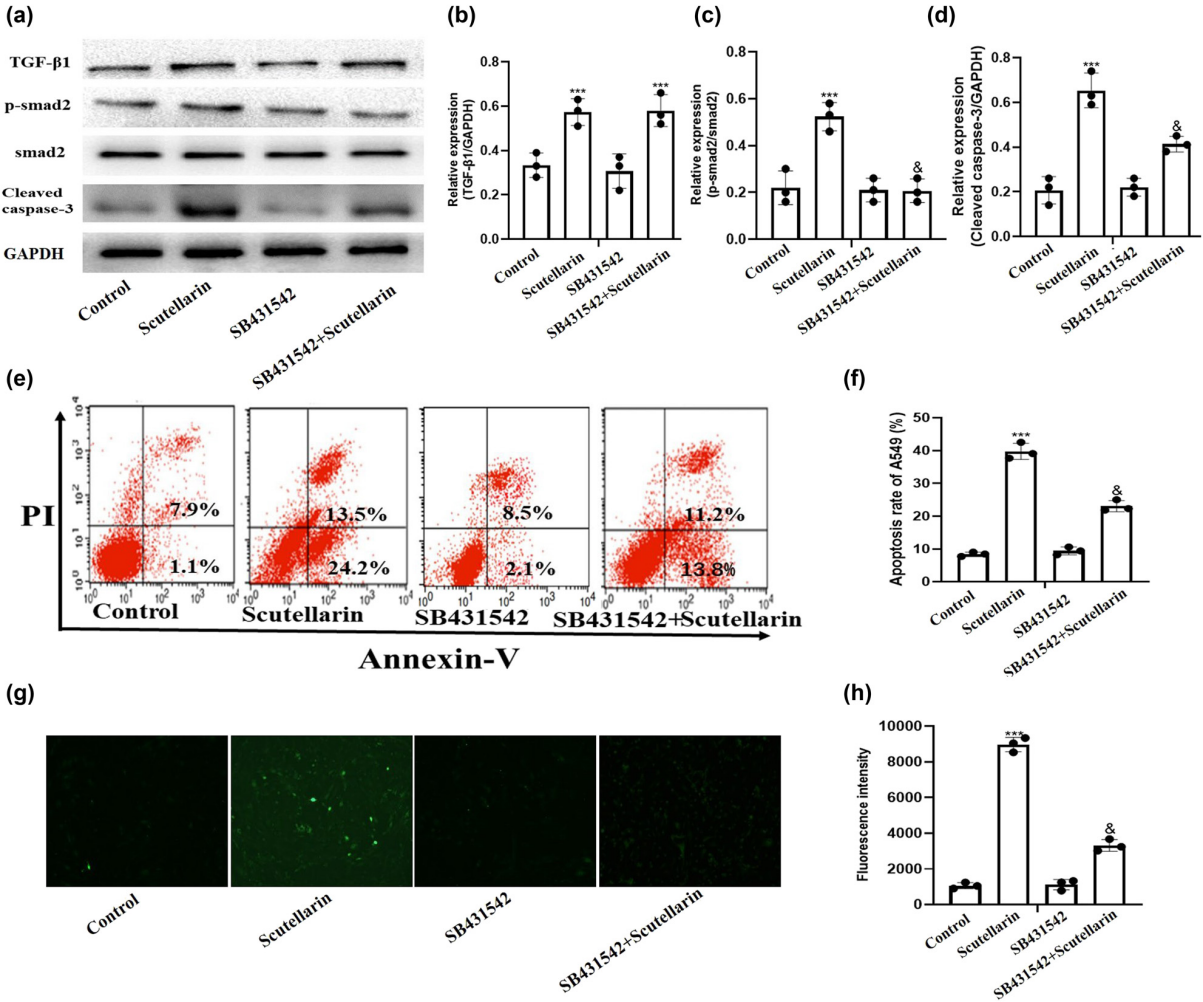
Intracellular ROS level and apoptosis of A549 cells induced by scutellarin were significantly decreased by blocking the TGF-β1/smad2 pathway with SB431542. (a–d) A549 cells were treated with 500 µM of scutellarin with and without 10 mM of SB431542 for 48 h and then the expressions of TGF-β1, p-smad2, and cleaved caspase-3 were examined in western blots. (e and f) Apoptosis of A549 cells was tested by flow cytometry and analyzed. (g and h) The intracellular ROS level was detected using a DHE fluorescence probe and then analyzed. ***P < 0.001 (scutellarin group vs control group) and *P* < 0.05 (scutellarin + SB431542 group vs scutellarin group).

We next examined whether ROS and TGF-β1/smad2 induced apoptosis through independent or similar mechanisms. SB431542, a TGF-β1 receptor inhibitor, was added to A549 cells before scutellarin treatment to block the TGF-β1 receptor. SB431542 inhibited scutellarin-induced TGF-β1/smad2 signaling pathway activation and cleaved caspase-3 expression (Figure 4a–d). SB431542 treatment also inhibited apoptosis in scutellarin-treated A549 cells (Figure 4e and f) and ROS accumulation (Figure 4g and h).

## 4 Discussion

In the present study, we discovered that scutellarin decreased the viability and enhanced the apoptosis of A549 cells in a dose- and time-dependent manner. Our data analysis showed that scutellarin also significantly increased the intracellular ROS level and cleaved caspase-3 production, which could be abrogated by Tempo treatment. Moreover, blocking the TGF-β1/smad2 pathway with SB431542 reversed cleaved caspase-3 expression, intracellular ROS accumulation, and apoptosis of A549 cells.

Scutellarin is a flavonoid isolated from the traditional Chinese medicine Erigeron breviscapus, which displays activity against cancer cells [17,18]. Cao et al. [19] reported that scutellarin plays a role in inhibiting proliferation and promoting apoptosis in A549 cells. Sun et al. [20] reported that scutellarin induced apoptosis of lung cancer cells *in vitro* and *in vivo*. It has also been reported that scutellarin ameliorated the drug resistance of A549/DDP cells to cisplatin by promoting apoptosis [21]. In our study, we found that scutellarin significantly suppressed the proliferation of A549 cells in a dose- and time-dependent manner and promoted cell apoptosis.

TGF-β is dysregulated in cancer, and as an important growth factor plays a crucial role in regulating tissue development and dynamic balance [22]. TGF-β exerts tumor-suppressive functions primarily resulting in apoptosis in the early phase of tumorigenesis [23]. TGF-β activates or inhibits the transcription of target genes through binding to its ligand and activating smads in the nucleus [24,25]. Studies have reported that TGF-β1 regulates apoptosis by activating smad2 [16,26,27]. Pan et al. [28] revealed that scutellarin suppressed TGF-β1 production to alleviate cardiac dysfunction of infarct rats. However, the effect of scutellarin on expression of TGF-β1 in cancer cells has not yet been examined. Our study observed that scutellarin activates the TGF-β1/smad2 signaling pathway and promotes the expression of cleaved caspase-3 in A549 cells.

Intracellular ROS induces DNA damage and cell apoptosis [29]. Scutellarin has different effects on the accumulation of ROS in normal cells and tumor cells. Scutellarin reduced apoptosis and ROS production to protect cardiomyocyte ischemia-reperfusion injury [30]. However, scutellarin treatment promoted apoptosis in multiple myeloma cells by inducing ROS accumulation [7]. Our study observed that intracellular ROS levels and the apoptosis rate were significantly increased in A549 cells after scutellarin exposure. We also observed that scutellarin induced upregulation of intracellular ROS levels and expression of cleaved caspase-3 were significantly inhibited by Tempo, a ROS scavenger.

TGF-β serves as a mediator of intracellular ROS production. Veith et al. [31] reported that TGF-β1 induced mitochondrial ROS production in human bronchial epithelial cells, which subsequently contributed to the epithelial injury and fibrotic lung scarring. TGF-β1 promoted the invasion and migration of cervical cancer cells by activating NOX4 to generate ROS production [32]. Our study provides evidence that blocking the TGF-β1/smad2 pathway significantly inhibited the scutellarin-induced increase in intracellular ROS levels and apoptosis in A549 cells. Taken together, these data suggest that the TGF-β1/smad2 pathway was activated by scutellarin, which subsequently induced the production of ROS and eventually resulted in A549 cell apoptosis.

To our knowledge, this study is the first to demonstrate that the TGF-β1/smad2/ROS/capsase-3 signaling pathway is involved in scutellarin-induced apoptosis of A549 cells. Considering its anti-tumor effect on tumor cells and its protective effect in normal tissue cells, scutellarin may be a promising anti-tumor agent in the future.
